# Comparative analysis of the mechanical behavior and plastic deformation of empty and polyurethane foam-filled polyethylene tubes under lateral compression

**DOI:** 10.1038/s41598-025-29184-y

**Published:** 2025-12-24

**Authors:** Seyedahmad Taghizadeh, Abbas Niknejad, Lorenzo Maccioni, Franco Concli

**Affiliations:** 1 Faculty of Science and Technology, Free University of Bozen-Bolzano, Bolzano, Italy; 2 Mechanical Engineering Department, Yasouj University, Yasouj, Iran

**Keywords:** Polyethylene tubes, Plastic deformation, Energy absorbing, Lateral compression, Polyurethane foam, Energy science and technology, Engineering, Materials science

## Abstract

In this research, the energy absorption and plastic deformation of circular polyethylene tubes under lateral compression were investigated, comparing empty tubes with those filled with polyurethane foam. The effects of tube length, diameter, wall thickness, and filler density on load-bearing capacity and energy absorption were evaluated. Absorbed energy and lateral load were found to increase with tube length, although the ductile response of polyethylene produced pronounced fluctuations in the load–displacement curve. Diameter influenced the two configurations differently: in empty tubes, smaller diameters yielded greater energy absorption, whereas in foam-filled tubes, larger diameters increased the flattening load and total absorbed energy, underscoring the critical role of diameter in foam-filled systems. Increasing wall thickness enhanced energy absorption, but polyurethane-foam filling was more effective and yielded a more consistent, predictable load–displacement response. Foam-filled tubes outperformed empty tubes overall; dominant damage mechanisms were identified as foam fracture, crushing, and densification. For applications prioritizing structural efficiency and specific absorbed energy (SAE), the use of higher-density polyurethane foam is recommended. Adhesion between the foam and the tube’s inner surface significantly increased peak load capacity. After relaxation, a uniform diameter reduction of approximately 12.7% was observed across specimens, indicating consistency in the final morphology. These findings provide guidance for the design of load-bearing systems employing polyethylene tubes and polyurethane foam.

## Introduction

Thin-walled structures are extensively employed in automotive, marine, and aerospace applications because of their high strength-to-weight ratios and capacity for controlled energy absorption. Considerable research has examined the crashworthiness of both empty and foam-filled tubes, consistently highlighting the influence of material selection and filler-tube interaction on deformation modes and energy-dissipation mechanisms. In particular, foam-filled configurations have demonstrated superior performance compared with empty tubes, as the synergistic action between tube walls and polymeric foams enhances load-bearing capacity, stabilizes deformation, and improves overall energy-absorption efficiency^1–5^. The energy-absorption characteristics of tubular structures with various geometries have been widely investigated, demonstrating effective dissipation of impact energy. Numerical studies of tubular members under axial loading have further clarified deformation patterns and failure mechanisms, reinforcing their utility in engineering design. Moreover, investigations of novel tubular structures with negative Poisson’s ratios have revealed improved mechanical properties, broadening potential applications in advanced engineering fields^6–8^. Circular tubes are especially recognized for their energy-absorption capability in crashworthiness applications. Multicell thin-walled tubes reinforced with topology-optimized lattice structures have been reported to achieve enhanced performance under both axial and lateral loading^9^. In addition, optimization of axial thickness variation in laterally corrugated tubes has been shown to significantly improve energy-absorption efficiency^10^. Mesoscale analyses of aluminum foam-filled carbon fiber-reinforced polymer (CFRP) tubes under axial compression have revealed substantial gains in mechanical properties and energy absorption^11^. An analytical model for the large-deflection behavior of foam-filled Y-shaped sandwich beams under lateral loads was developed by Jianxun Zhang et al.^12^, incorporating stretching-bending interactions; numerical simulations validated the model and demonstrated strong sensitivity to metal foam strength, face-sheet thickness, and punch width. The lateral compression behavior of thin-walled equilateral triangular tubes has also been examined through quasi-static tests^13^, wherein three crushing stages were identified, and plastic models were proposed to predict collapse behavior. Symmetrical crushing modes in ideal tubes were highlighted, and structural strengths were accurately predicted by balancing yielding and buckling, indicating unified energy-absorption mechanisms governed by multiple plastic hinges. The mechanical properties and failure behavior of advanced carbon/carbon (C/C) composite tubes intended for nuclear reactor structures have been investigated using quasi-static compression tests to characterize load–displacement responses and damage evolution; acoustic emission and imaging were employed to monitor crack initiation and propagation, and finite element simulations were used to analyze stress distributions^14^. Strong energy-absorption capacity and stable load-bearing performance were demonstrated, supporting optimization of nuclear structural designs. A simplified model consisting of elastic panels and nonlinear springs was proposed by Sohrab Defaei and Hossein Ezati Asl^15^ to analyze steel infill panels; by concentrating inelasticity at discrete connections, the approach reduced computational cost while maintaining accuracy. Validation against six experimental steel shear panels showed reliable prediction of global response and failure patterns, and the model’s simplicity facilitates implementation in standard engineering software. The response of 3D6d-braided composite tubes to lateral compression was investigated by Xiaoxu Wang et al.^16^, with emphasis on the influence of the diameter-to-thickness (D/t) ratio on damage progression; the crucial role of sixth-directional yarns in enhancing structural performance was confirmed. The mechanical behavior of thin-walled tubes subjected to lateral compression was also explored by Ziqian Zhang^17^ using finite element analysis with experimental validation; a theoretical model was introduced to predict cross-sectional deformation in the large elastic–plastic stage, together with an innovative solution strategy for integrals with variable upper limits, and the model’s range of applicability was delineated. A novel cenosphere/aluminum syntactic foam exhibiting high strength and energy absorption was presented by Boyi Zhang et al.^18^; deformation of foam-filled tubes under lateral compression was analyzed with a focus on the six-plastic-hinge mode, and the effects of cenosphere size and tube wall thickness on overall absorption were quantified. Related work on brass and composite tubes has examined the influence of polyurethane foam filling under lateral compression, showing higher energy absorption and reduced risks of fiber fracture and circumferential delamination compared with empty tubes^19–21^. A substantial body of work has thus addressed tubular structures under lateral loading, considering configuration, material systems, and loading scenarios. Experimental studies have been widely performed to observe deformation patterns, failure modes, and load–displacement relationships under lateral compression^22–25^. Nevertheless, despite this breadth of research, the mechanical response of polyethylene tubes under lateral compressive loads remains relatively underexplored. Empty polyethylene tubes are widely employed in water and oil transportation due to their corrosion resistance and durability, yet a clear understanding of their failure mechanisms and the governing parameter under lateral compression is needed to ensure reliability in demanding environments.

Flattening behavior of circular polyethylene tubes, in empty and polyurethane-foam-filled conditions, is investigated experimentally under controlled variations in tube length, diameter, wall thickness, and foam density. The fraction of recoverable elastic energy within the total absorbed energy is quantified. The effect of post-test relaxation time on the stabilized post-compression geometry is characterized. The specific contribution of filler-tube adhesion to the lateral response is isolated. Taken together, these results establish polyethylene tubes as tunable energy absorbers and provide concrete design guidance for lateral compression.

## Experimental procedure

**Fig. 1:**
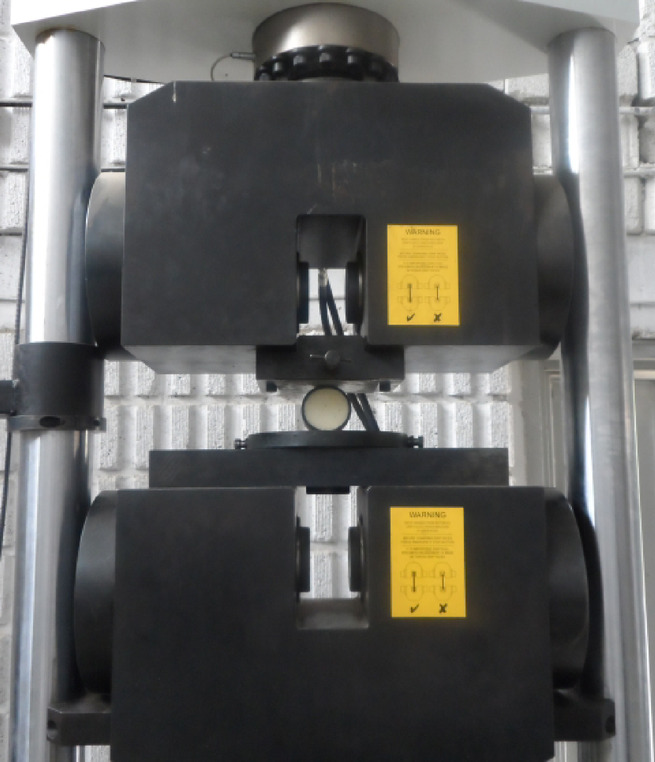
The specimen G-02 in the testing machine.

Lateral compression tests were performed on circular polyethylene tubes, in both empty and polyurethane-foam-filled conditions, using a DMG testing machine. During flattening, the tubes were placed between rigid plates to ensure uniform loading. To examine the effects of tube diameter, length, and wall thickness on absorbed energy and lateral load, seven geometric groups of specimens were prepared; each group comprised tubes with identical diameter and wall thickness but different lengths. Within each group, two empty specimens and one polyurethane-foam-filled specimen of the same dimensions were tested to assess the influence of foam filling on lateral load capacity and energy absorption. Initial measurements of diameter, length, and wall thickness were recorded before testing. The tubes were then positioned horizontally between the machine’s rigid plates, and compression was applied at a constant displacement rate until complete flattening was achieved. Lateral load and displacement were recorded continuously for subsequent determination of energy absorption and peak lateral load. A foam-filled circular polyethylene tube mounted in the apparatus before compression is shown in Fig. 1. All tests were performed in triplicate, and repeated load–displacement measurements on the same specimen varied by less than 1.5 percent, indicating excellent repeatability; accordingly, absorbed energy for each test was computed as the area under the recorded load–displacement curve.

The geometric specifications of the empty circular polyethylene tubes are presented in Table 1, while Table 2 provides the corresponding details for the foam-filled specimens. Tube length, external diameter, wall thickness, and mass are denoted by *L*, *D*, *t*, and *m*, respectively. Each flattening test was conducted at a velocity of 2 mm/min under quasi-static conditions. Three distinct densities and corresponding plateau stresses were employed for the polyurethane foam fillers, as summarized in Table 3. The foams were produced from polyol and isocyanate components, with mixing ratios adjusted to obtain different target densities. To characterize their compressive behavior, cubic specimens (1 cm3) were tested under out-of-plane quasi-static compression in accordance with ASTM D1621, and the resulting densities and plateau stresses were used in the analysis. Additionally, in specimen F-08, the polyurethane foam was pre-expanded outside the tube and inserted without adhesion, whereas in all other foam-filled specimens, the foam was injected directly into the tubes, allowing for in-situ expansion and natural adhesion to the inner tube walls. This study aims to investigate the influence of adhesion on lateral load capacity and energy absorption by comparing two identical foam-filled specimens, F-06 and F-08, fabricated with and without adhesion, respectively.

**Table 1:** Mass and geometric characteristics of empty tubes.

Specimen code	D (mm)	t (mm)	L (mm)	m (g)
A-01	20	1.9	40	4.67
A-03	20	1.9	30	3.49
A-04	20	1.9	20	2.34
B-01	25	1.9	50	7.46
B-03	25	1.9	30	4.55
B-04	25	1.9	25	3.84
C-01	40	2	40	9.5
C-03	40	2	30	7.23
C-04	40	2	20	5.15
D-01	50	3	50	25.27
D-03	50	3	30	14.58
D-04	50	3	25	12.89
E-01	63	3	63	36.32
E-03	63	3	30	17.05
E-04	63	3	31.5	19.92
F-01	63	5	63	51.21
F-03	63	5	30	24.63
F-04	63	5	31.5	27.04
G-01	78	4	78	71.61
G-03	78	4	30	28.11
G-04	78	4	39	35.76

**Table 2:** The polyurethane foam-filled tubes’ geometric measurements and mass.

Specimen code	D (mm)	t (mm)	L (mm)	m (g)	Foam-filler
A-02	20	1.9	30	5.1	Foam01
B-02	25	1.9	30	8.2	Foam01
C-02	40	2	30	14.21	Foam01
D-02	50	3	30	24.59	Foam01
E-02	63	3	30	33.49	Foam01
E-05	63	3	30	24.12	Foam02
E-06	63	3	30	21.73	Foam03
F-02	63	5	30	42.17	Foam01
F-05	63	5	63	63.48	Foam03
F-06	63	5	30	31.12	Foam03
F-07	63	5	31.5	31.24	Foam03
F-08	63	5	30	29.52	Foam03
G-02	78	4	30	51.19	Foam01

**Table 3:** lists the characteristics of the polyurethane foam fillers.

Specimen code	Density (kg/m^3^)	Plateau stress (MPa)
Foam01	256	1.76
Foam02	84.8	0.57
Foam03	55.55	0.09

## Results and discussion

The deformation modes, failure mechanisms, and collapse behavior of circular polyethylene tubes during lateral flattening were investigated experimentally. The effects of geometric parameters-length, wall thickness, and diameter on the tube performance were examined. In addition, the roles of foam-filler properties-density, adhesion, and the presence of filler on lateral force, total absorbed energy, and specific absorbed energy were analyzed.

### Effects of tube length

**Fig. 2:**
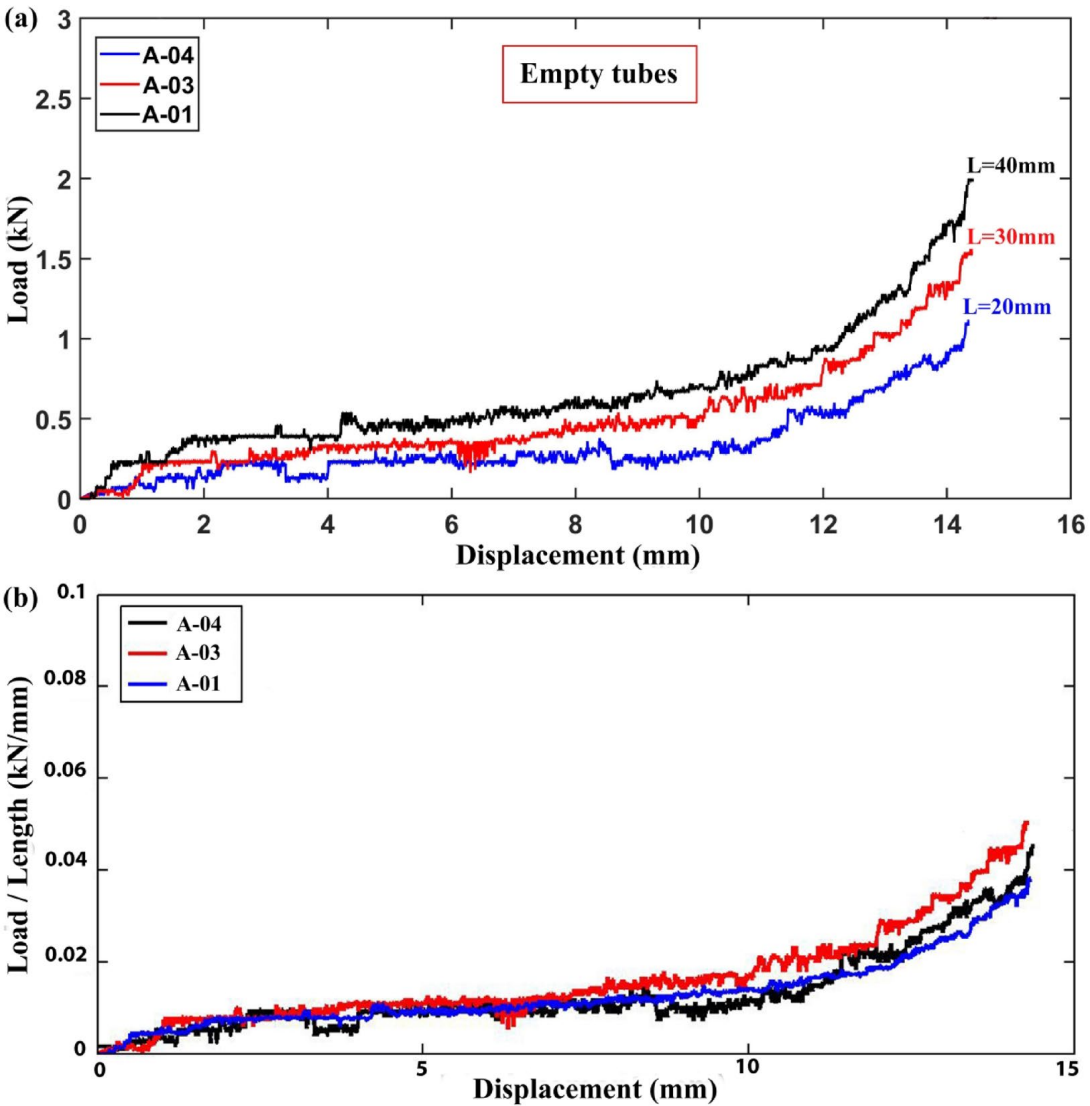
Lateral compressive load per unit length versus displacement for empty specimens (all samples: t = 1.9 mm, D = 20 mm).

The load–displacement and load/length-lateral displacement graphs for specimens with different lengths but the same diameter and wall thickness are shown in Fig. 2. The figures demonstrate the proportional connection between lateral load and the empty tube length and confirm the reliability of test findings by comparing lateral load per unit length versus lateral displacement. The ratios of lengths A-03/A-01 and A-04/A-01 equal 1.5 and 2.0, respectively, according to experimental measurements. The lateral load of specimens A-01, A-03, and A-04 is 639 N, 941 N, and 1120 N, respectively, corresponding to a lateral displacement of 12.6 mm. In accordance, these specimens’ absorbed energies up to an axial displacement of 12.6 mm are 3.315 J, 5.162 J, and 7.006 J, in that order. As such, the axial load ratios for A-03/A-01 and A-04/A-01 are 1.473 and 1.753, respectively, while the absorbed energy ratios are 1.492 and 2.025 for A-03/A-01 and A-04/A-01, respectively. These findings support a direct connection between tube length, absorbed energy, and lateral load. Due to the ductile nature of polyethylene, the load–displacement curve exhibits noticeable fluctuations. Polyethylene’s inherent ductility enables it to undergo substantial deformation before failure, causing the load–displacement curve to display multiple fluctuations and troughs as the material yields and undergoes strain hardening during lateral compression.

**Fig. 3:**
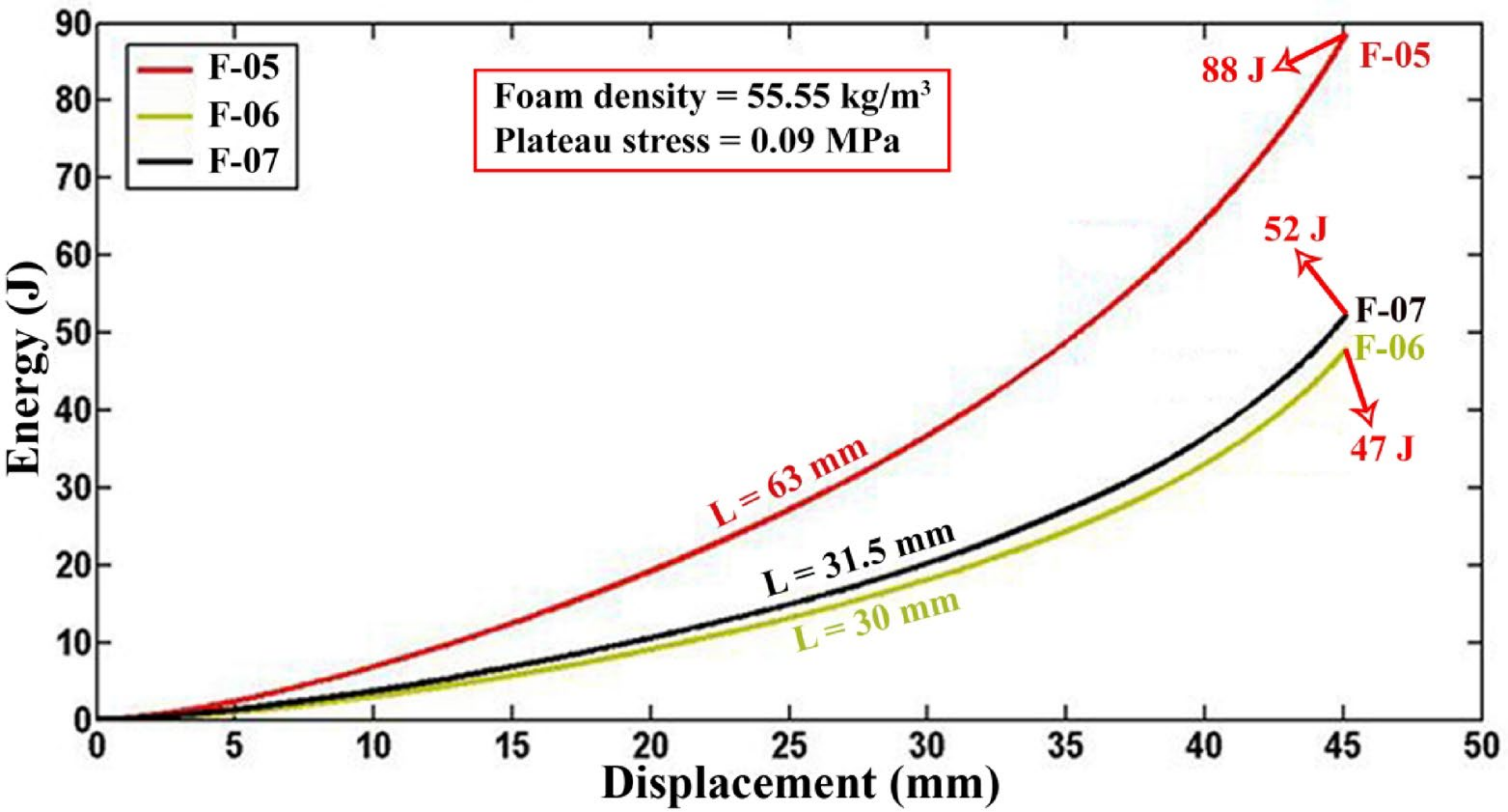
Energy-displacement curves of foam-filled specimens F-05, F-06, and F-07 with varying lengths but identical other characteristics (t = 5 mm, D = 63 mm).

In Fig. 3, the absorbed energy-displacement curves compare polyurethane foam-filled specimens of varying lengths but with consistent other characteristics. For specimens F-05, F-06, and F-07 filled with polyurethane foam, the absorbed energies up to a 40 mm lateral displacement are reported as 64.563 J, 33.084 J, and 36.481 J, respectively. The experimental results show that the absorbed energy ratios for F-05/F-06 and F-07/F-06 are 1.952 and 1.1026, respectively, with corresponding length ratios of 2.1 and 1.05. These findings illustrate a clear linear correlation between absorbed energy in foam-filled tubes and tube length.

### Effects of tube diameter

**Fig. 4:**
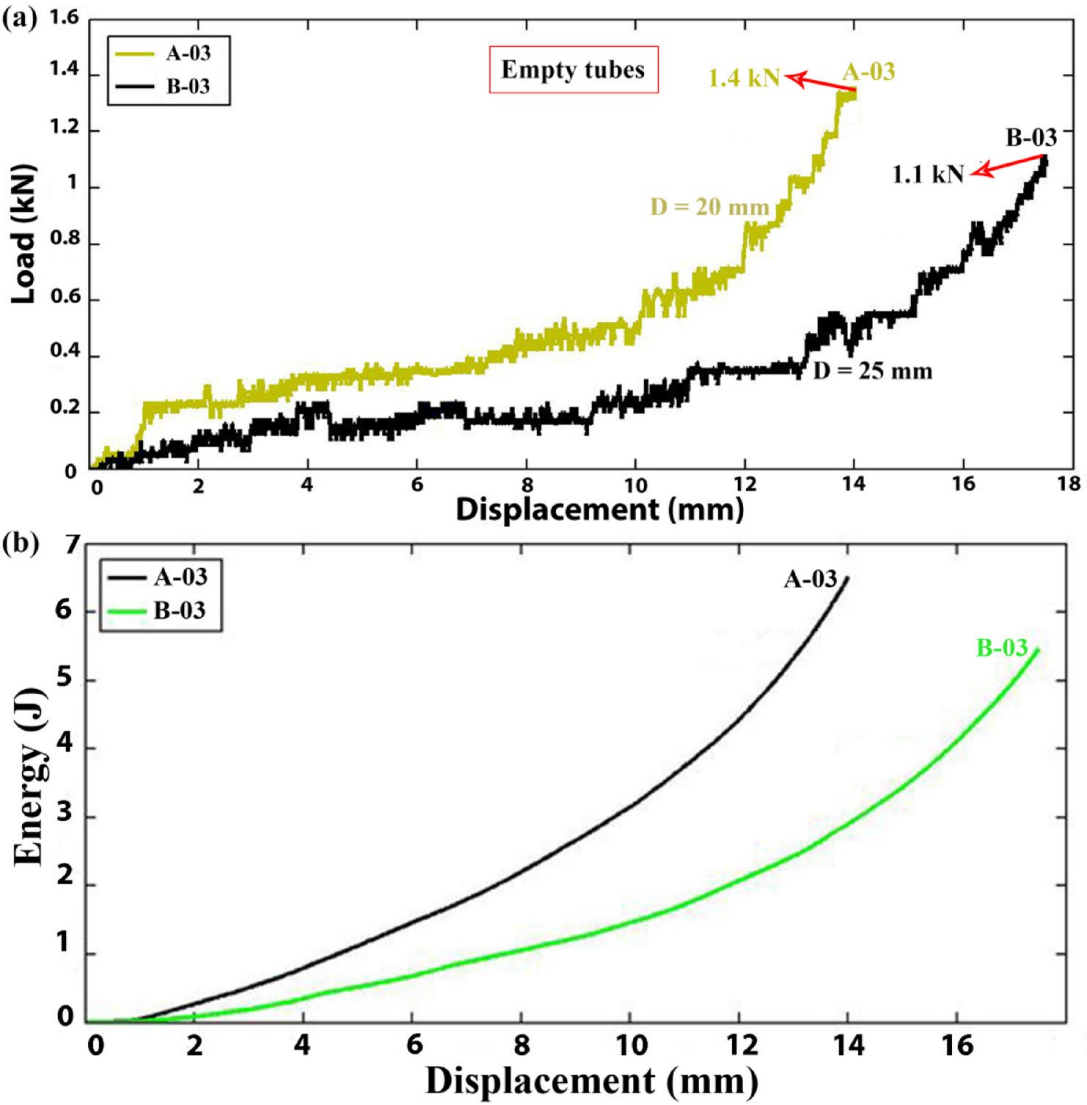
Load and energy versus displacement of the empty tubes with different diameters (t = 1.9 mm, L = 30 mm).

Figure 4a illustrates the impact of tube diameter on the instantaneous and mean lateral loads of specimens A-03 and B-03, characterized by a consistent length of 30 mm and tube wall thickness of 1.9 mm, but differing external diameters of 20 mm and 25 mm, respectively. The graph demonstrates that specimen A-03 exhibits a higher instantaneous lateral load compared to B-03. Additionally, Fig. 4b presents an experimental diagram depicting the absorbed energy of specimens A-03 and B-03 as a function of lateral displacement during lateral compression tests, normalized by their respective lateral displacement-to-diameter ratio of 0.7. The results show that at specific lateral displacements, the tube with the larger diameter absorbs less energy compared to the tube with the smaller diameter. This trend also holds true for the total absorbed energy by the empty tubes. Therefore, from the perspective of designing energy absorption systems, tubes with smaller diameters are preferable over tubes with larger diameters, all else being equal, due to their enhanced energy absorption capabilities.

**Fig. 5:**
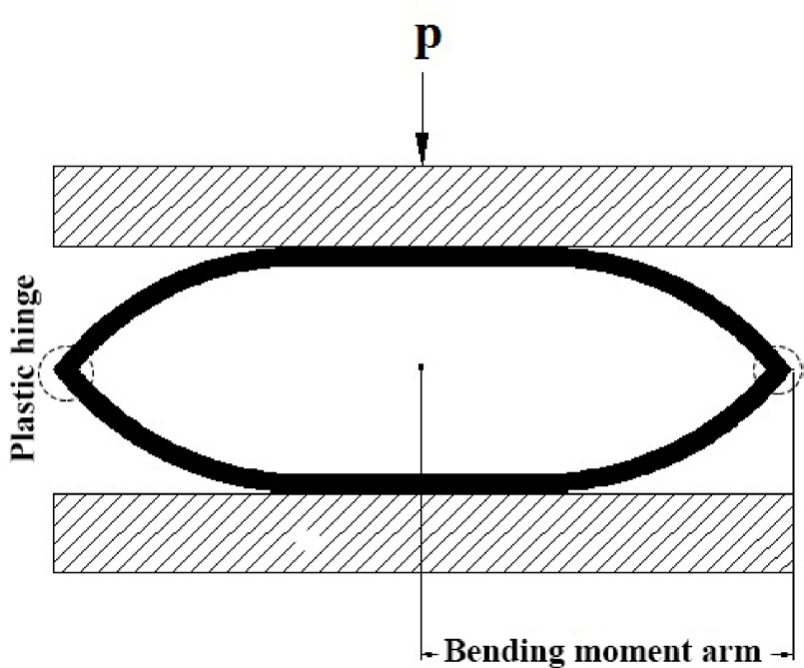
Bending moment arm around the plastic hinge lines.

Experimental observations indicate that, during the flattening of empty polyethylene tubes between two rigid platens, two plastic hinge lines are formed along the left and right sides of the tube cross-section, as schematically depicted in Fig. 5.

**Fig. 6:**
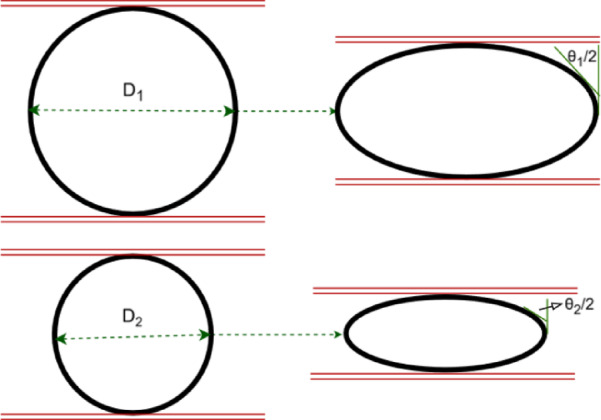
Schematic comparison of the angle of θ at the same lateral displacement in empty tubes with different diameters.

According to Fig. 6, the lateral resultant load is applied on the upper and lower points of the tube’s external surface. Therefore, as shown in Fig. 5, an increment of the tube’s external diameter leads to an increase of the bending moment arm, and it causes the lower required lateral load to flatten the empty tube. The following relation can estimate the summation of the absorbed energies by the plastic deformation in two plastic hinge lines:

1 $$E = 2M_{0} L\theta$$

Here, *L* represents the tube’s length, *θ* denotes the rotation angle around each plastic hinge line, and *M*_0_ refers to the fully plastic bending moment per unit length of the tube, calculated as follows:

2 $$M_{0} = \frac{1}{4}\sigma_{0} t^{2}$$

In the above equation, *t* represents the tube’s initial wall thickness, while *σ*_0_ denotes the flow stress of the tube material, which is estimated using the following relation^26^:

3 $$\sigma_{0} = \sqrt {\frac{{\sigma_{y} \cdot \sigma_{u} }}{1 + n}}$$

Here, *σ*_γ_, *σ*_γ_, and *n* denote the yield stress, ultimate stress, and strain hardening exponent, respectively. By substituting Eq. (2) into Eq. (1), the following expression is obtained for estimating the energy absorbed through plastic deformation at the two plastic hinge lines:

4 $$E = \frac{1}{2}\sigma_{0} t^{2} L\theta$$

During the flattening process of circular polyethylene tubes, the angle *θ* increases from zero to *π*. Consequently, the total energy absorbed by the plastic hinge lines of the empty tubes throughout the flattening process is determined as follows:

5 $$E = \frac{\pi }{2}\sigma_{0} t^{2} L$$

Equation (4) demonstrates that the energy absorbed through plastic deformation at the two plastic hinge lines is directly proportional to the tube length. As illustrated in Fig. 6, at a given lateral displacement, the rotation angle *θ* in a tube with a larger diameter is smaller than that in a tube with a smaller diameter. Consequently, as per Eq. (1), the absorbed energy of a larger-diameter tube is lower than that of a smaller-diameter tube.

**Fig. 7:**
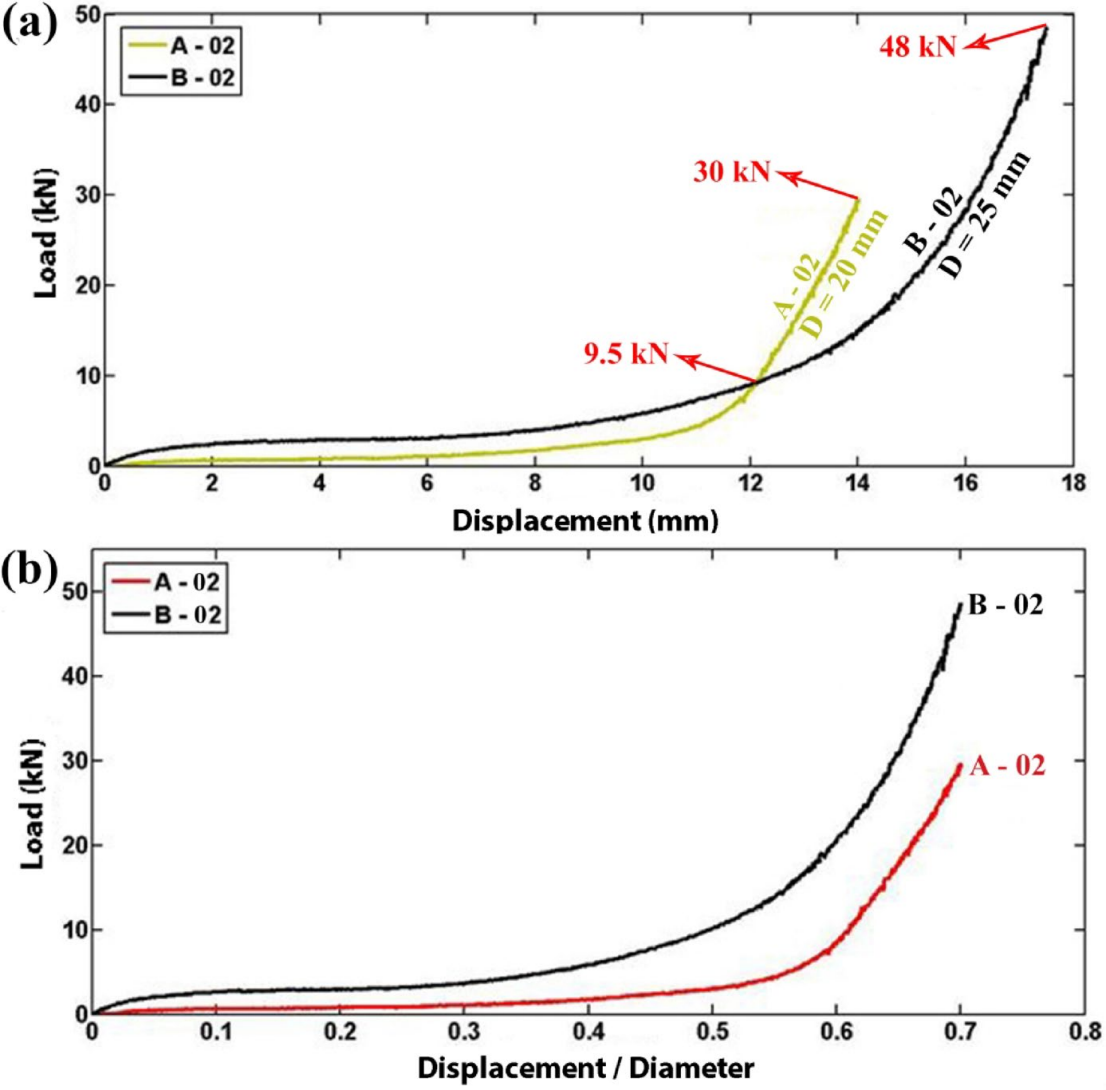
Diagram of the foam-filled tubes with the different diameters (t = 1.9 mm, L = 30 mm), (**a**) lateral load–displacement curves, (**b**) lateral load versus the displacement/diameter ratio.

In Fig. 7a, the lateral load–displacement responses of the polyurethane foam-filled specimens A-02 and B-02 are compared. These specimens share the same length, wall thickness, and material composition for both the tubes and the foam but differ in their internal diameters. To provide a more detailed comparison, Fig. 7b presents a plot of the lateral load as a function of the lateral displacement-to-inner diameter ratio for these specimens. The experimental findings indicate that, for the polyurethane foam-filled specimens, an increase in tube diameter results in higher flattening loads and, consequently, greater energy absorption capacity at the same displacement-to-diameter ratio.

**Fig. 8:**
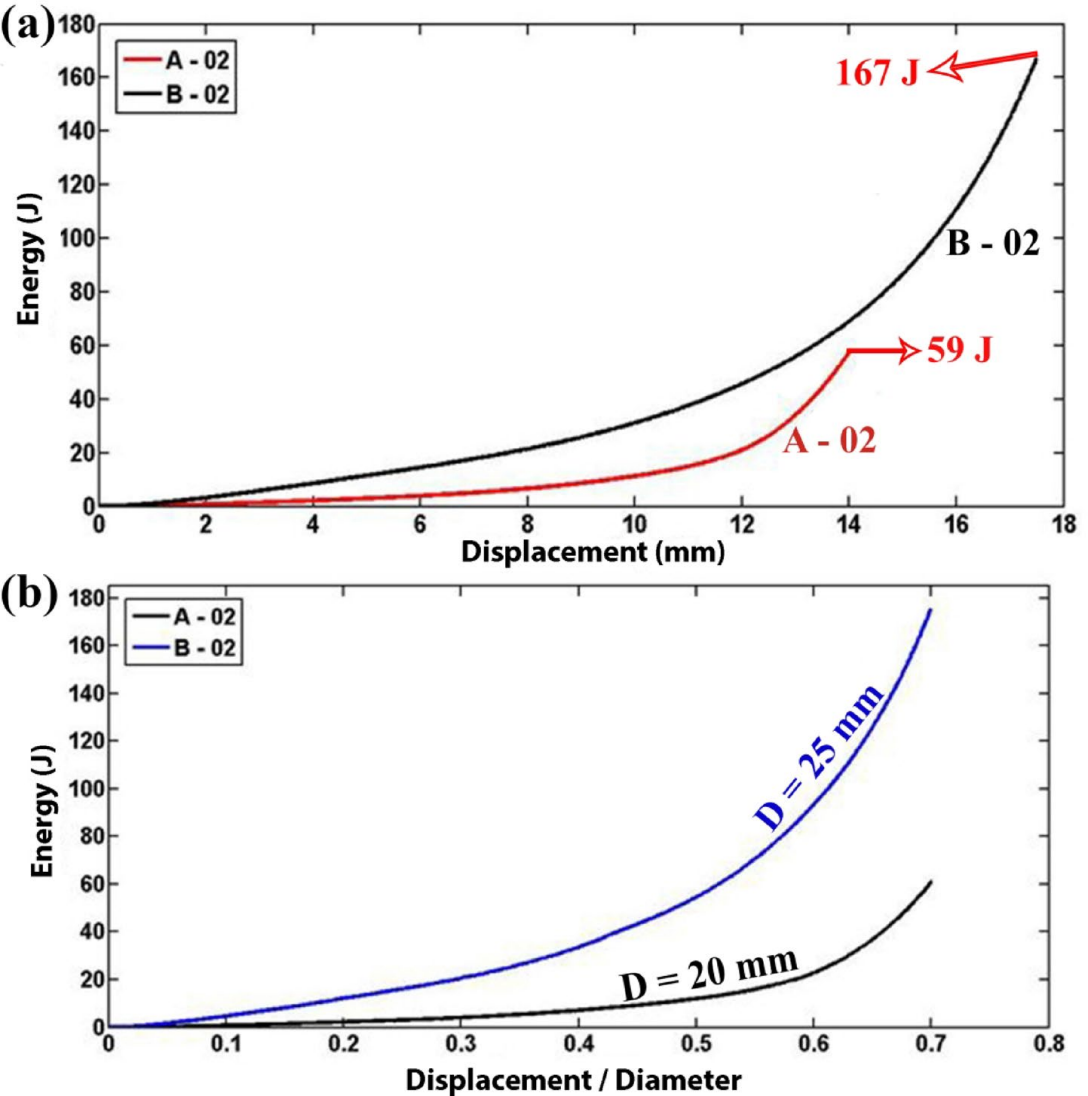
Comparison of absorbed energy in foam-filled tubes with varying diameters (t = 1.9 mm, L = 30 mm): (**a**) absorbed energy as a function of displacement and (**b**) absorbed energy as a function of the displacement-to-diameter ratio.

Figure 8a and b present diagrams of the absorbed energy of the polyurethane foam-filled specimens as a function of lateral displacement and displacement-to-inner diameter ratio, respectively. The results reveal that for the foam-filled specimens A-02 and B-02, both with a length of 30 mm, a wall thickness of 1.9 mm, and external diameters of 20 mm and 25 mm, respectively, the instantaneous flattening load and energy absorption are greater in specimen B-02. This enhancement is ascribed to the larger external diameter, which results in an augmented volume of polyurethane foam filler, thereby amplifying the proportion of energy dissipated through foam deformation.

**Fig. 9:**
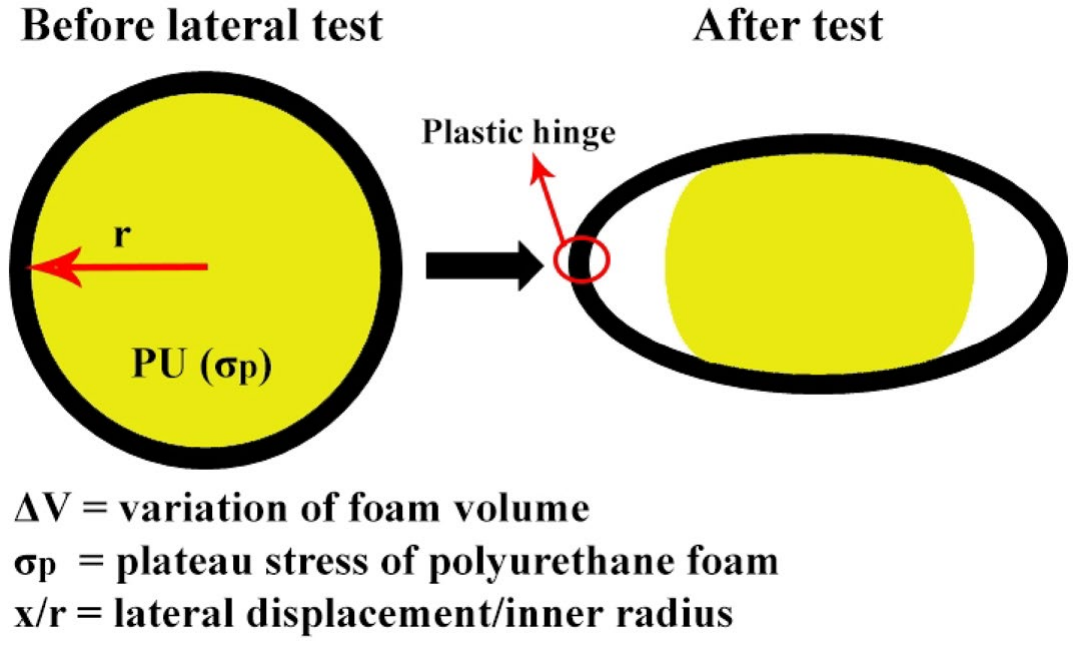
Theoretical deformation model illustrating the flattening behavior of a foam-filled specimen.

**Fig. 10:**
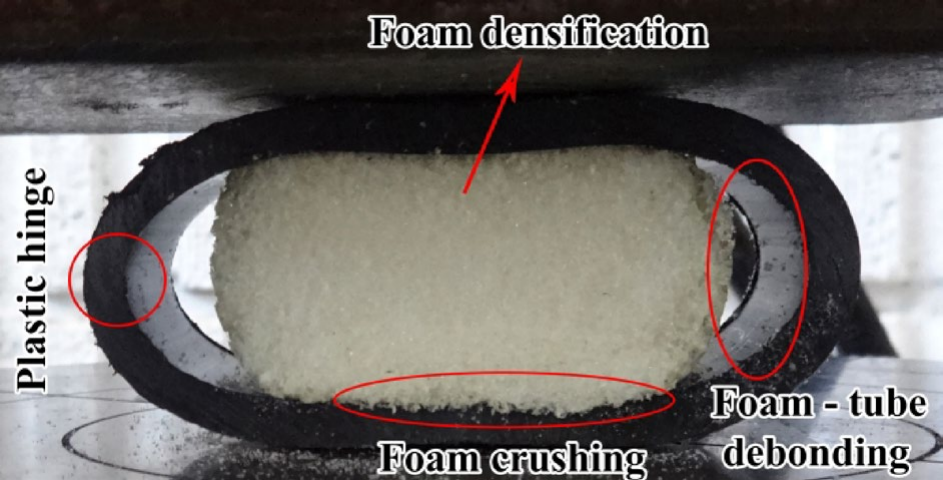
The specimen F-06 after the flattening test.

Figure 9 shows a theoretical deformation model of foam-filled polyethylene tubes with initial circular cross-section before and during the flattening process. For a better conclusion, Fig. 10 illustrates the foam-filled specimen F-06 during the flattening test. Compression tests on the foam and, on the foam-filled specimens show that the Poisson’s effect on the polyurethane foam can be neglected and therefore, the Poisson’s ratio of the polyurethane foam can be considered equal to zero. Therefore, according to the introduced theoretical model of deformation for the foam-filled polyethylene tubes during the flattening process in Fig. 10, the hatched area is calculated as:

6 $$\Delta A = r^{2} (\alpha - \sin \alpha )$$

In Fig. 9, the angle *α* is visible, where *r* denotes the inner radius of the tube, and *d* = *2r* represents the inner diameter. The preceding equation calculates the change in the cross-sectional area of the polyurethane foam (ΔA) during lateral compression. Referring to Fig. 9, the lateral displacement can be expressed as:

7 $$x = r(1 - \cos \frac{\alpha }{2})$$

Calculating the value of angle α from Eq. (7) and substituting in Eq. (6) results in:

8 $$\Delta A = 2r^{2} \left\{ {\cos^{ - 1} (1 - \frac{x}{r}) - (1 - \frac{x}{r}) \cdot \sin \left[ {\cos^{ - 1} (1 - \frac{x}{r})} \right]} \right\}$$

​The energy absorbed by polyurethane foam during compression is generally calculated as:

9 $$E_{foam} = \sigma_{p} \cdot \Delta V = \sigma_{p} \cdot \Delta A \cdot L$$

In the above relation, ΔV indicated the variation of the foam volume during the plastic deformation. σ_p_ denotes the plateau stress of polyurethane foam. Substituting Eq. (8) in Eq. (9) results in the following relation:

10 $$E_{foam} = 2r^{2} L\sigma_{p} \cdot \left\{ {\cos^{ - 1} (1 - \frac{x}{r}) - (1 - \frac{x}{r}) \cdot \sin \left[ {\cos^{ - 1} (1 - \frac{x}{r})} \right]} \right\}$$

The energy absorbed by polyurethane foam as a function of lateral displacement, x, during the flattening process is estimated using Eq. (10). Based on this theoretical relationship, for foam-filled tubes with varying inner diameters (d = 2r) but identical other characteristics, the energy absorbed by polyurethane foam with a larger initial diameter is greater than that absorbed by foam with a smaller initial diameter when the same ratio of lateral displacement to inner radius (x/r) is considered. This results in an increased energy absorption capacity for foam-filled polyethylene tubes with larger diameters. Experimental findings indicate that the total absorbed energies for the empty and foam-filled specimens A-03 and A-02, up to a lateral displacement-to-diameter ratio of 0.7, are 6.78 J and 61.20 J, respectively. Consequently, the contribution of polyurethane foam to the total absorbed energy is significantly greater than that of the tube itself. Therefore, the hierarchy of absorbed energy in foam-filled polyethylene tubes with different diameters is determined by the energy absorbed by the polyurethane foam alone.

### Effects of tube wall thickness

**Fig. 11:**
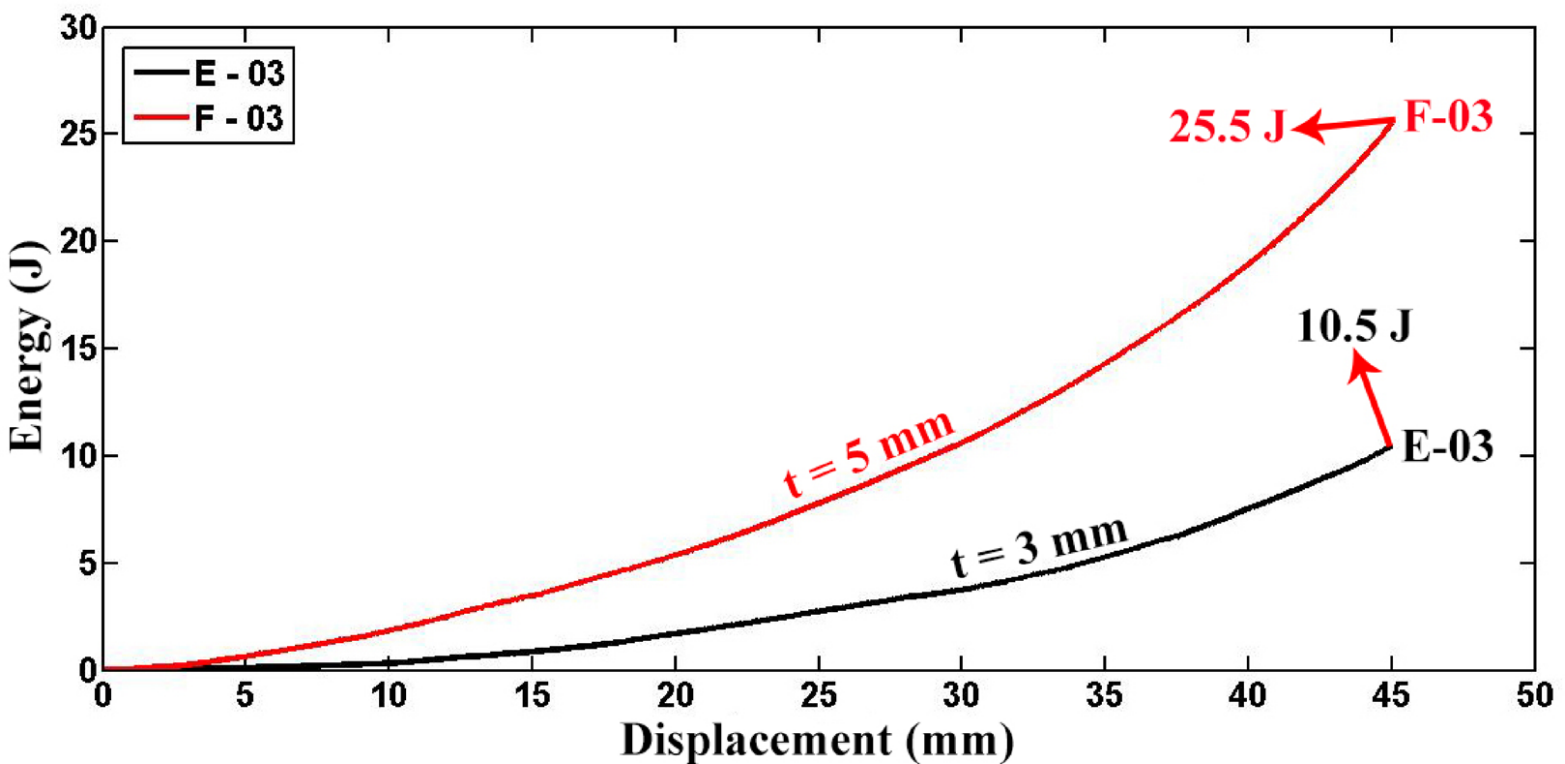
Absorbed energy versus displacement for empty specimens F-03 and E-03, with different wall thicknesses (D = 63 mm, L = 30 mm).

Figure 11 illustrates the absorbed energy-displacement diagram for empty specimens E-03 and F-03. Both specimens share a length of 30 mm and an external diameter of 63 mm but differ in wall thicknesses of 3 mm and 5 mm, respectively. As depicted in the figure, specimen F-03 demonstrates higher energy absorption capability compared to specimen E-03. The ratio of wall thicknesses between specimens F-03 and E-03 is 1.667, with total absorbed energies up to a lateral displacement of 45 mm recorded as 10.479 J and 25.503 J, respectively, resulting in an absorbed energy ratio of 2.434 for specimens F-03/E-03. According to Eq. (5), which relates absorbed energy during plastic deformation in the plastic hinge lines of an empty tube to the square of its radius, an increase in wall thickness by a factor of 1.667 theoretically predicts a 2.779 times increase in energy dissipation. The experimental ratio of 2.434 aligns reasonably well with this prediction, indicating that a significant portion of the absorbed energy in empty polyethylene tubes is dissipated through plastic deformation in the plastic hinge lines. This suggests that Eq. (5) provides a useful initial estimate for absorbed energy during lateral flattening of polyethylene tubes. Moreover, the experimental findings affirm that increasing tube wall thickness enhances energy absorption by circular tubes.

**Fig. 12:**
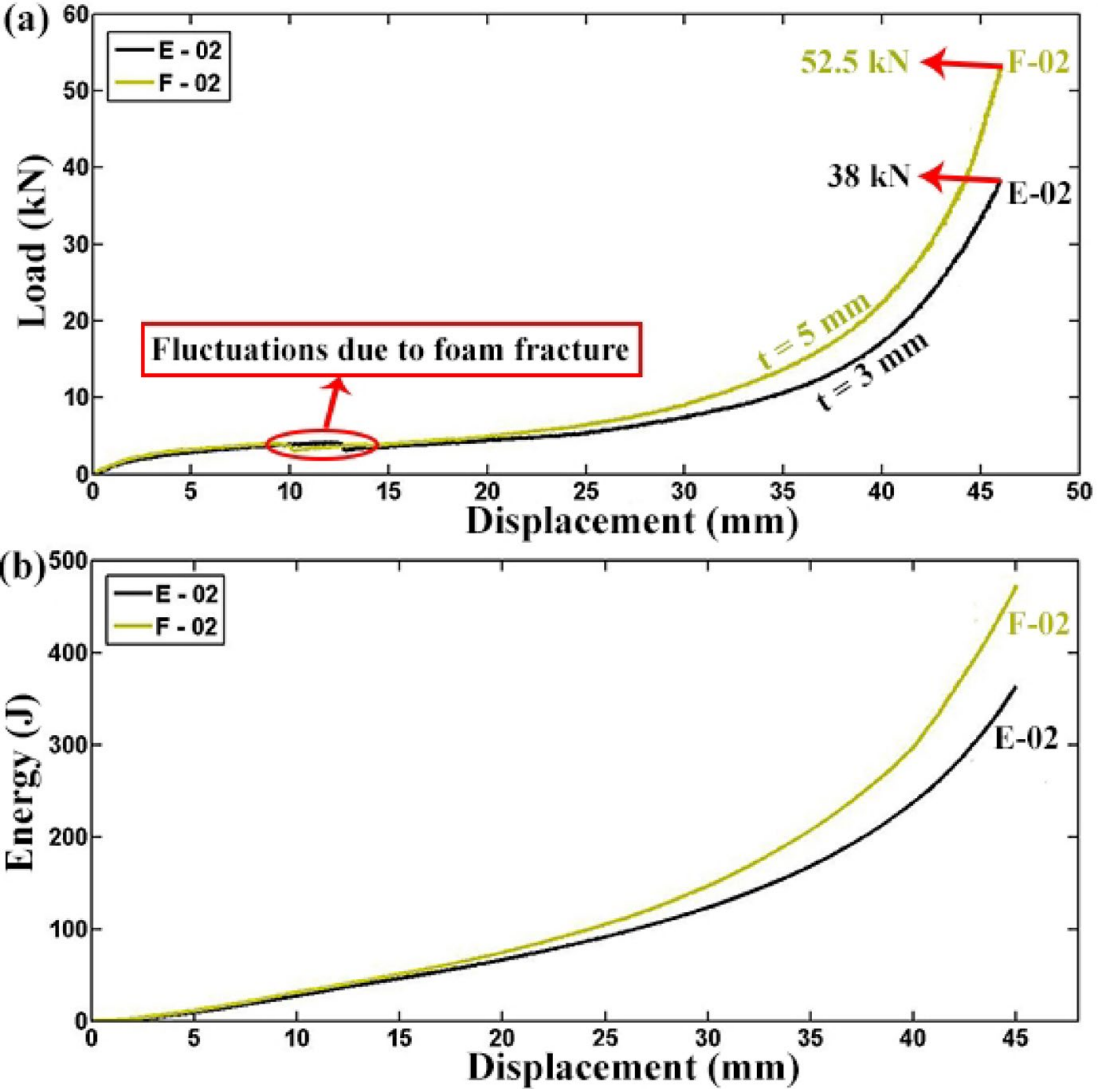
Comparison of experimental results for foam-filled specimens F-02 and E-02, each with a diameter of 63 mm and length of 30 mm but differing wall thicknesses: (**a**) load–displacement and (**b**) absorbed energy-displacement curves.

Figure 12 investigates the impact of tube wall thickness on polyurethane foam-filled tubes, presenting load–displacement and absorbed energy-displacement diagrams for specimens E-02 and F-02 with varying wall thicknesses but identical other characteristics. The experiments reveal that while the thicker-walled tube absorbs more energy than the thinner-walled one, their energy absorption capacities are less pronounced than empty tubes. For instance, the absorbed energies for filled specimens with 3 mm and 5 mm wall thicknesses are 363.496 J and 473.266 J, respectively, resulting in 1.302 times increase in absorbed energy for a 1.667 times increment in wall thickness. Thus, filling polyethylene tubes with polyurethane foam is preferable over thickening the tube wall when considering energy absorption capabilities. This preference is supported by the findings that increasing wall thickness does not significantly enhance energy absorption as effectively as filling the tube with foam.

### Effects of polyurethane foam-filler

**Fig.13:**
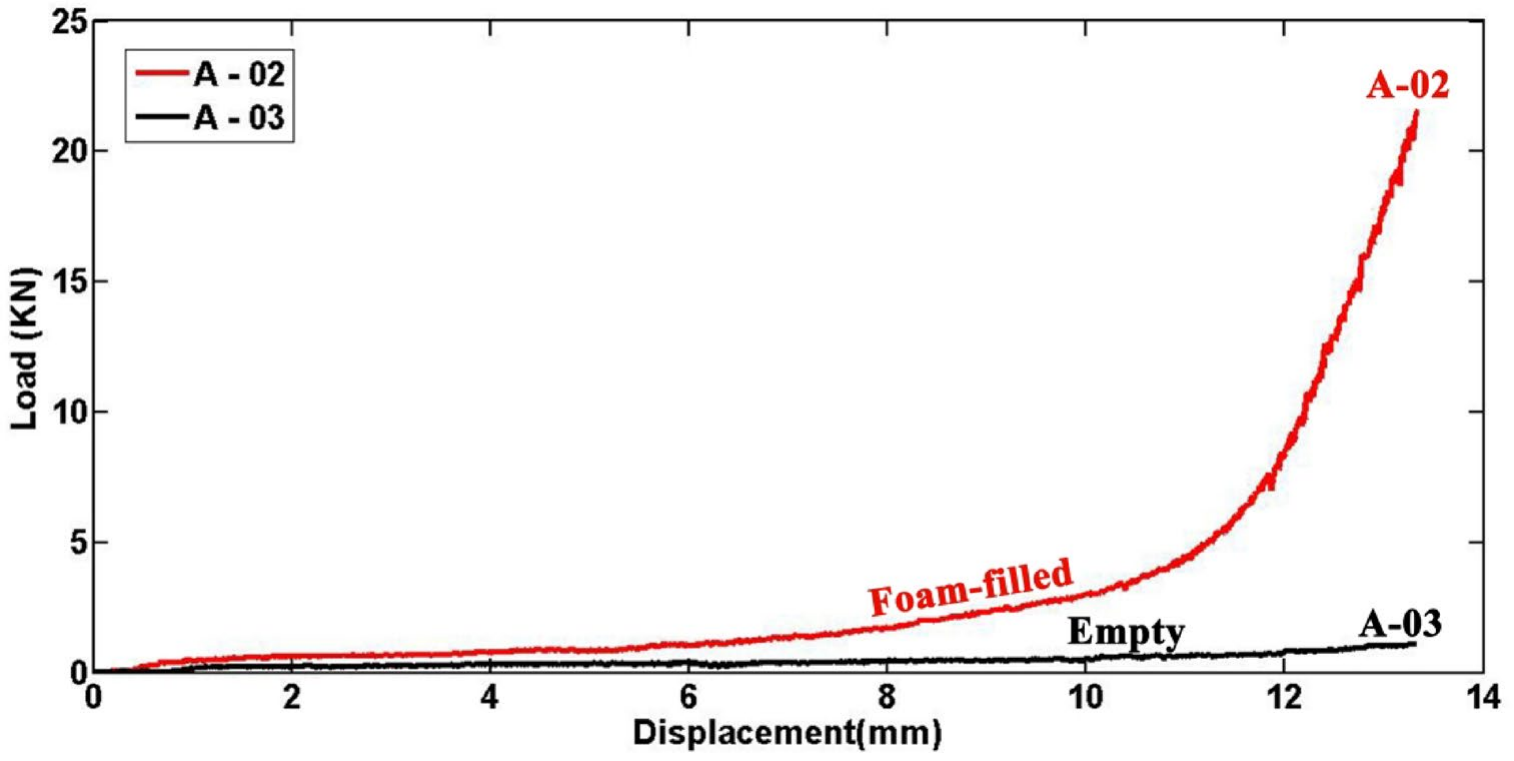
Load–displacement curves for empty and foam-filled specimens A-02 and A-03, each with a wall thickness of 1.9 mm, diameter of 20 mm, and length of 30 mm.

Polyurethane foam is widely recognized for its effectiveness in enhancing energy absorption in polyethylene circular tubes subjected to lateral compression. Figure 13 presents lateral load–displacement diagrams comparing an empty specimen, A-03, with a foam-filled specimen, A-02, both sharing identical dimensions of 30 mm length, 20 mm diameter, and 1.9 mm wall thickness. The diagram highlights that at a lateral displacement of 13.5 mm, the lateral forces are 22 kN and 1.4 kN for specimens A-02 and A-03, respectively. Moreover, experiments reveal that the absorbed energy for foam-filled specimen F-02 is 488.34 J, while the corresponding value for the empty specimen F-03 is 26.83 J. This indicates that filling the tubes with polyurethane foam increases lateral load and absorbed energy by factors of 38.778 and 18.201, respectively. Additionally, up to a displacement of 46.1 mm, the specific absorbed energy (absorbed energy per unit mass) for specimens F-02 and F-03 is 11.44 J/kg and 1.13 J/kg, respectively, showing that the foam-filled specimen F-02 absorbs 10.124 times more energy per unit mass than the empty specimen F-03. This substantial enhancement underscores the advantage of using polyurethane foam as a filler for creating efficient energy absorbers in polyethylene tubes. Comparing the Figs. 4, 7, and 8 reveals significant differences in the effects of tube diameter on lateral force and absorbed energy between empty and foam-filled tubes. In empty tubes, increasing diameter reduces lateral load and consequently lowers energy absorption, whereas in foam-filled tubes, larger diameters enhance energy absorption. This distinction underscores that while larger diameters are advantageous for foam-filled tubes, smaller diameters are preferable for empty tubes in energy absorption systems. Furthermore, comparing the lateral load–displacement diagrams of empty and foam-filled specimens shows that in foam-filled specimens, lateral load variations are more consistent and predictable compared to empty specimens.

**Fig. 14:**
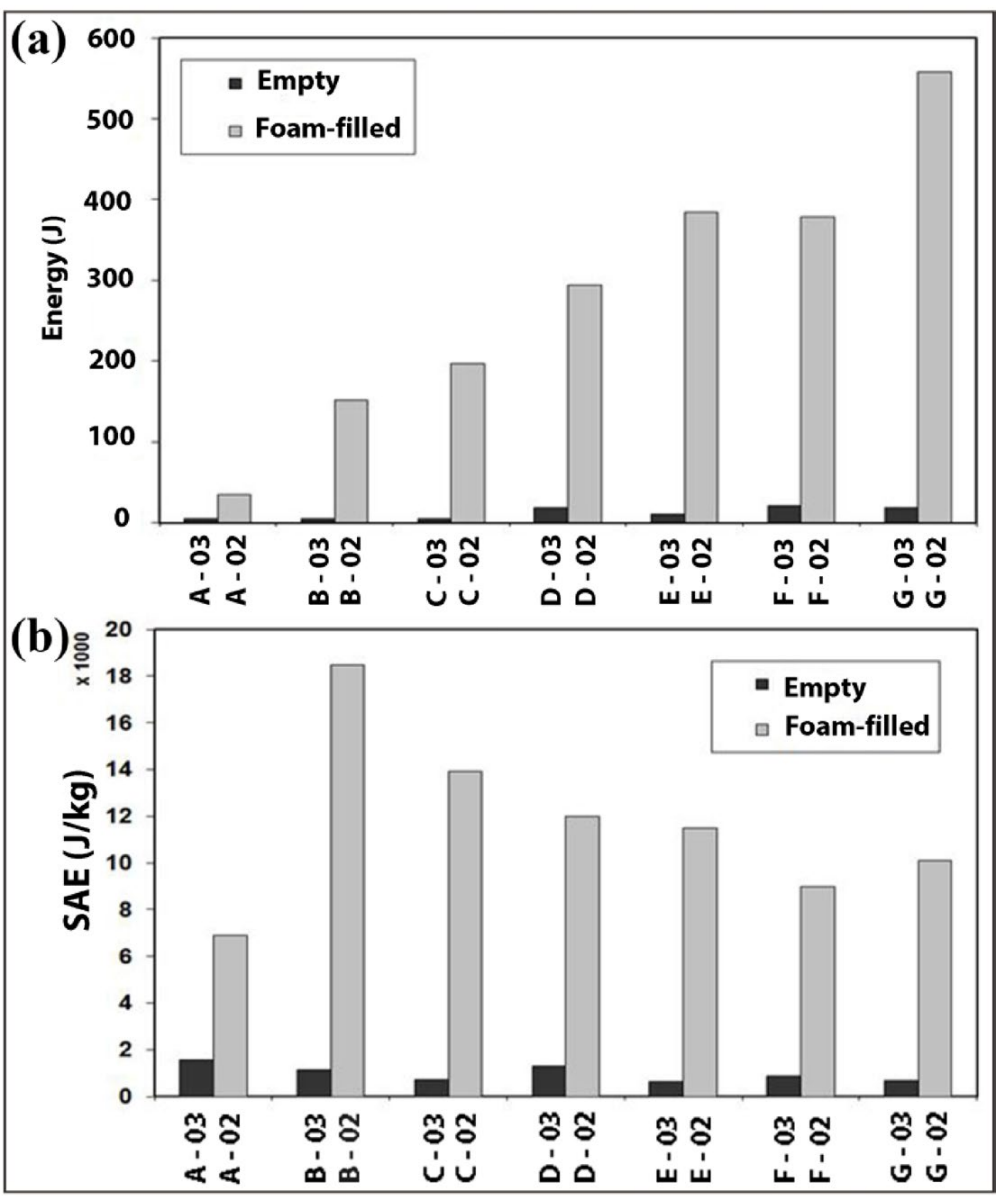
Evaluation of (**a**) total energy absorption and (**b**) specific energy absorption in empty and foam-filled specimens.

Figure 14a and b compare the absorbed energy and specific absorbed energy of both empty and foam-filled specimens during the flattening process. Figure 14a demonstrates that despite the thicker wall of foam-filled specimen F-02 compared to foam-filled specimen E-02, F-02 with an inner diameter of 53 mm absorbs less energy than E-02 with an inner diameter of 57 mm. Moreover, the difference in their specific absorbed energies is notable. Additionally, Fig. 14a highlights that foam-filled specimen G-02 exhibits the highest overall energy absorption capacity, while Fig. 14b indicates that foam-filled specimen B-02 achieves the highest specific absorbed energy (SAE). Furthermore, specimens D-02 and E-02, which share the same wall thickness and length but differ in diameter, show that E-02 absorbs more total energy than D-02. However, E-02 has a lower specific absorbed energy compared to D-02, which contrasts with findings from foam-filled specimens A-02 and B-02. These results underscore the existence of an optimal inner diameter for each selected wall thickness to maximize energy absorption per unit mass. Figure 14b also demonstrates that the highest SAE observed exceeds 18,000 J/kg, implying that one kilogram of an optimized polyethylene tube during flattening can effectively dissipate the kinetic energy equivalent to a 1,000 kg automobile traveling at an initial velocity of 21.6 km/h.

### Effects of foam density

**Fig. 15:**
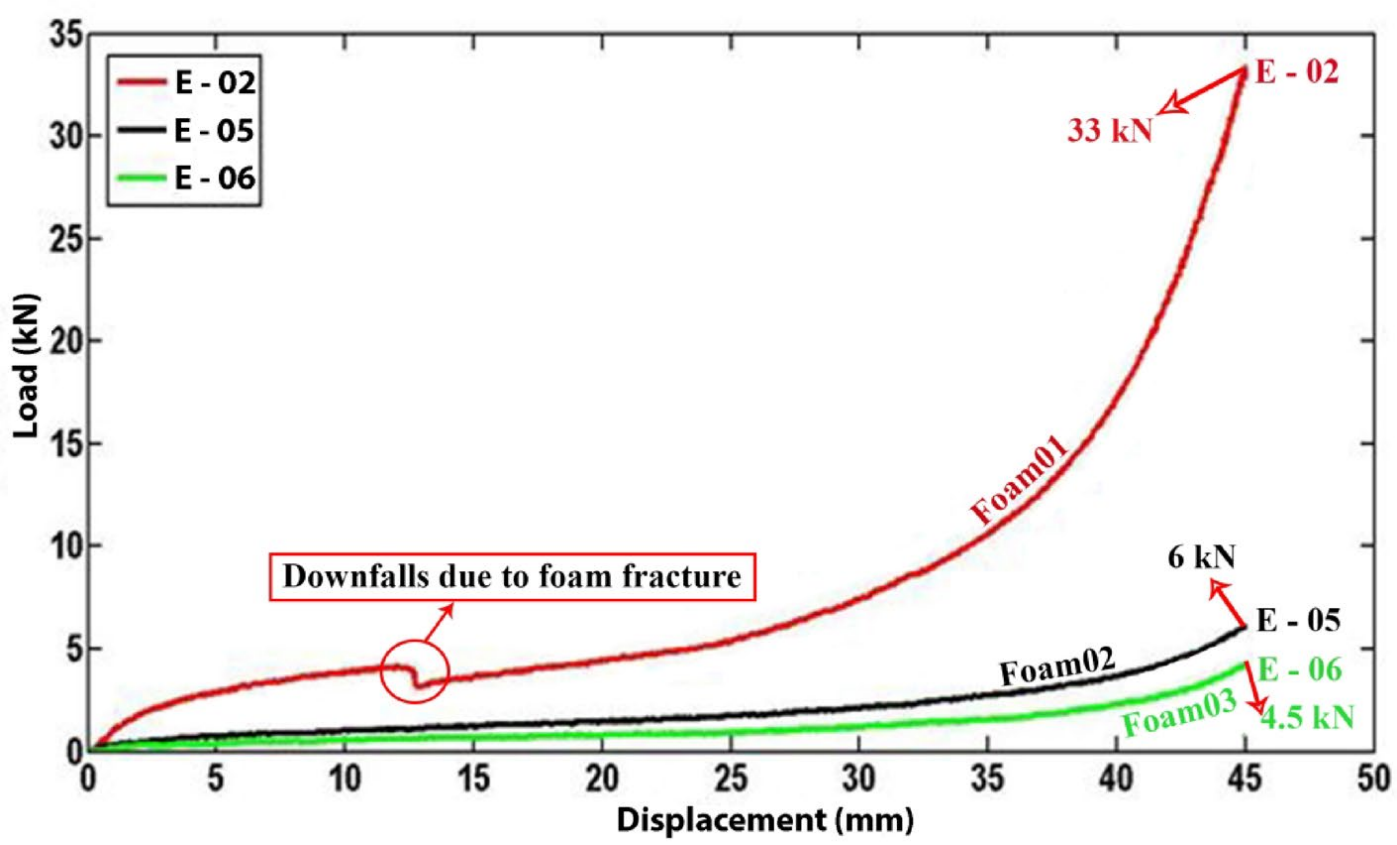
Diagram of the lateral load versus the lateral displacement of the same tubes with the different foam-fillers.

**Fig. 16:**
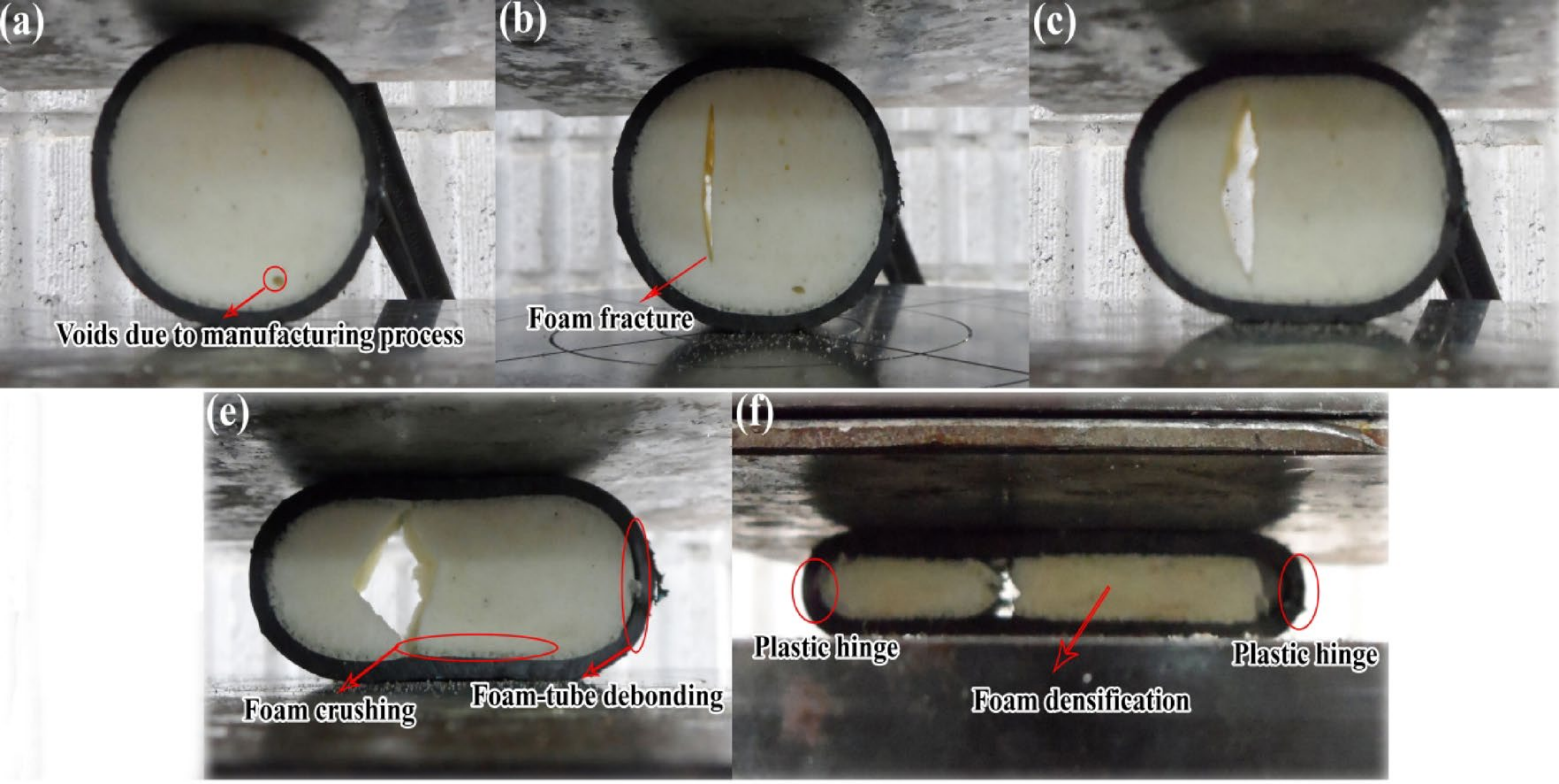
Failure and deformation modes of a foam-filled polyethylene tube during the flattening test.

Polyethylene tubes with uniform characteristics were filled with polyurethane foams of varying densities to assess the impact of foam density on the energy absorption capacity of the filled tubes. Figure 15 presents experimental data comparing specimens E-02, E-05, and E-06, filled with polyurethane foams of densities 256 kg/m3, 84.8 kg/m3, and 55.55 kg/m3, respectively. The data indicate that an increase in polyurethane foam density correlates with an increase in lateral load during the flattening process. Additionally, Fig. 16 reveals a significant decrease in the load–displacement curve for foam-filled specimen E-02, suggesting foam fracture during testing. This fracture is associated with a reduction in lateral load and may involve damage mechanisms such as foam crushing, foam-tube debonding, and foam densification observed during the flattening process.

**Fig. 17:**
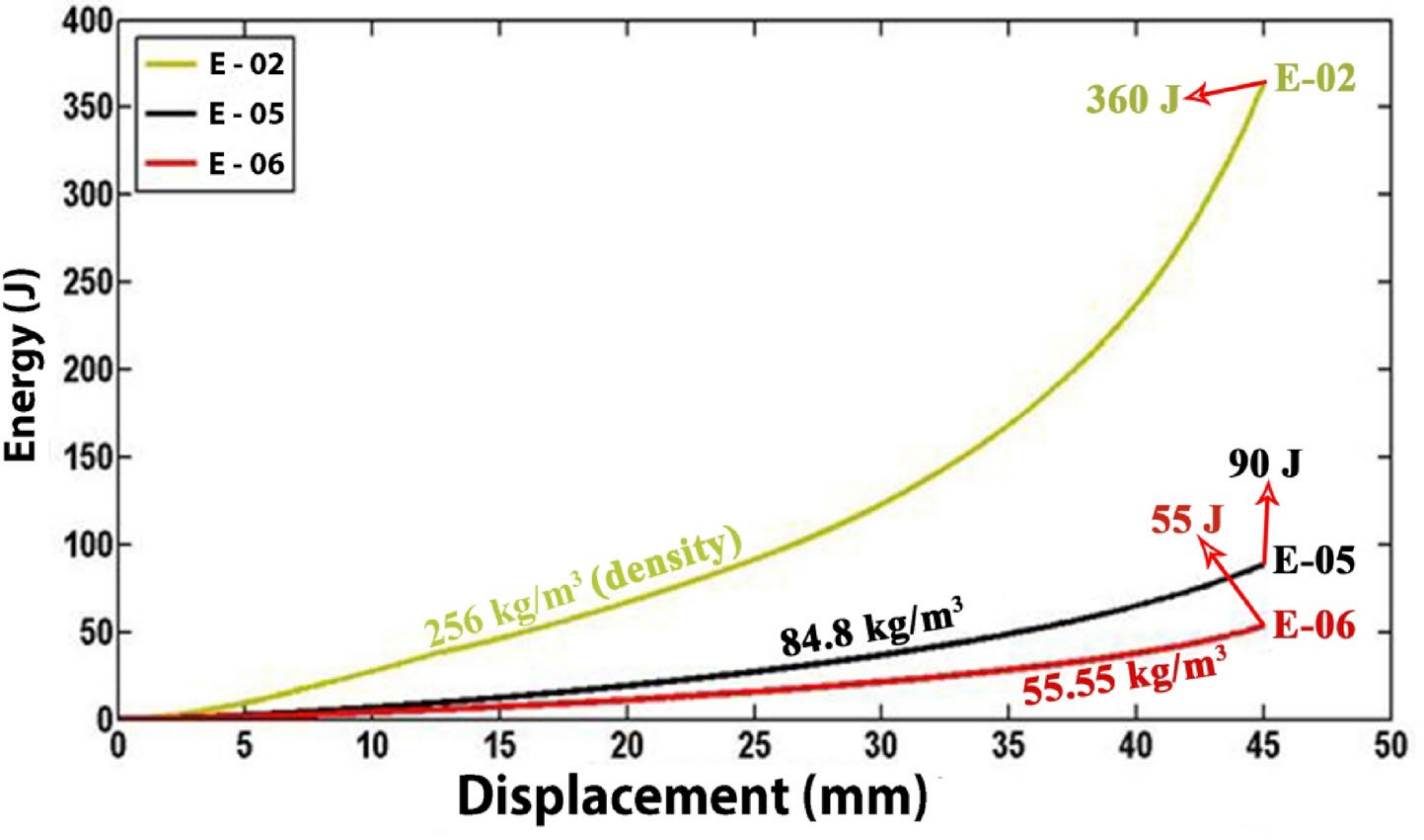
Effect of foam filler density on absorbed energy during lateral displacement of tubes.

Figure 17 depicts the energy absorbed by identical tubes containing foam fillers of varying densities during the flattening process. The data indicate that an increase in foam density results in a corresponding increase in the energy absorbed by the specimen. Specifically, the foam filler in specimen E-02 exhibits a higher density compared to specimens E-05 and E-06, leading to a significantly greater specific absorbed energy (SAE) in E-02 relative to E-05 and E-06. These findings suggest that, when both structural mass and SAE are critical parameters, the utilization of higher-density polyurethane foam is a prudent choice.

### Effects of relaxation time

**Fig. 18:**
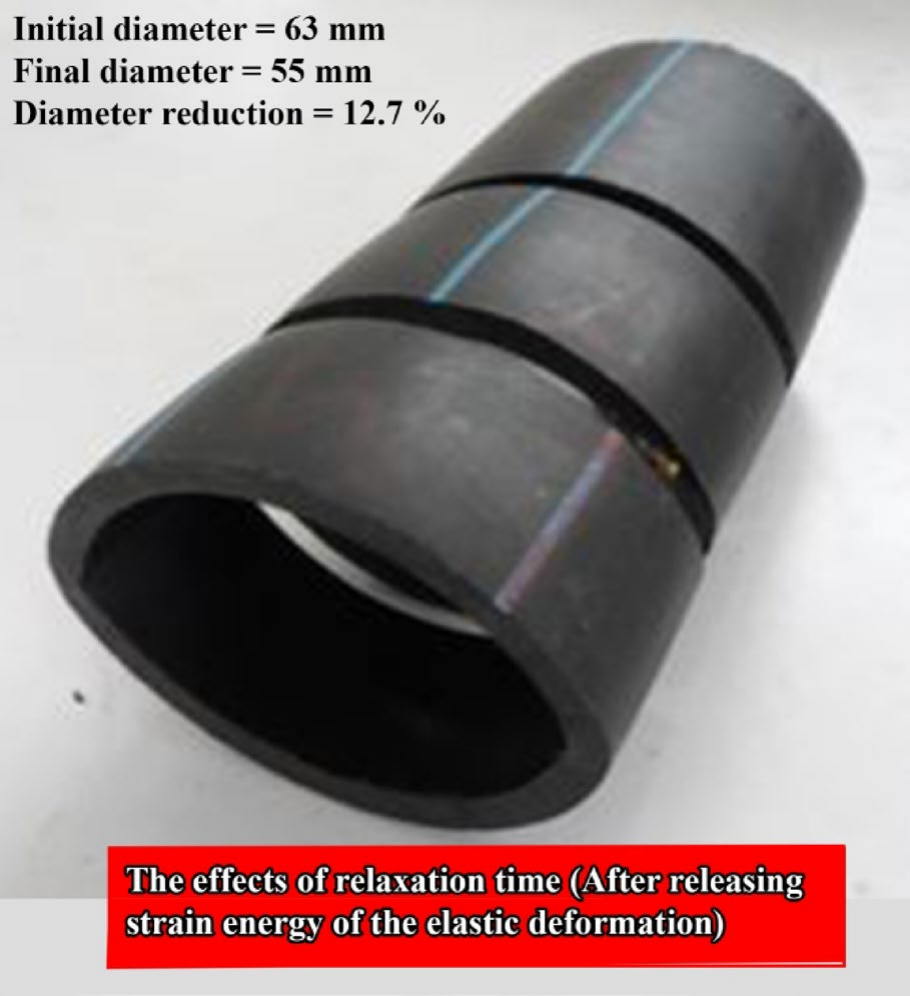
Final shape of the specimens F-01, F-03, and F-04 after the same period.

In this paper, for the first time, the effects of relaxation time on the final shape of the specimens after the flattening process are investigated. In other words, after the compression test, the strain energy of the elastic deformation is released and this can be observed in changes of the final shape of the specimens during a period of time after each test. Figure 18 shows the final shape of some empty specimens with different initial lengths and with the same other characteristics after the relaxation time. The figure shows that for the specimens with the same cross-section and material, the final shape of the specimens after the relaxation time is almost the same and the diameter reduction of the mentioned tubes after the relaxation time is equal to 12.7%, compared with the initial diameter.

**Fig. 19:**
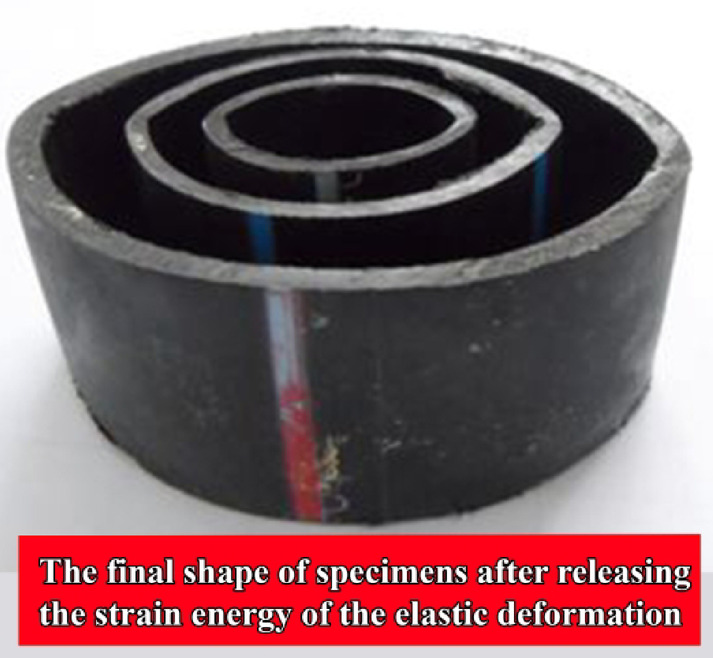
Final shape of the different tubes with the different initial geometrical dimensions.

Table 4 gives the diameters of the initial and the final shapes of the tube’s cross-section. The listed results in the table show that the final shape of the specimens is independent of the geometrical characteristics and the percentage of the diameter reduction for all specimens is approximately between 10 and 15%. For a better conclusion, Fig. 19 shows the final shape of the different tubes with different geometrical dimensions, and it shows a similar percentage of diameter reduction. Experimental observations show that after the tests, variations of the specimen’s shape decreased by increasing the relaxation time and, after a certain time (nearly 3 days), no changes were observed in the final shape of the specimen, because the elastic strain energy was completely released.

**Table 4:** Diameters of the initial and the final cross-section of the flattened specimens.

Specimen No	Initial diameter (mm)	Final diameter (mm)	Diameter reduction (%)
A—01	20	18	10
A—03	20	18	10
A—04	20	18	10
B—01	25	22	12
B—03	25	22	12
B—04	25	22	12
C—01	40	34	15
C—03	40	34	15
C—04	40	33	15
D—01	50	43	14
D—03	50	43	14
D—04	50	43	14
E—01	63	56	11
E—03	63	55	12.7
E—04	63	55	12.7
F—01	63	55	12.7
F—03	63	55	12.7
F—04	63	55	12.7
G—01	78	68	12.8
G—03	78	69	11.5
G—04	78	67	14.1

### Effects of adhesion

**Fig. 20:**
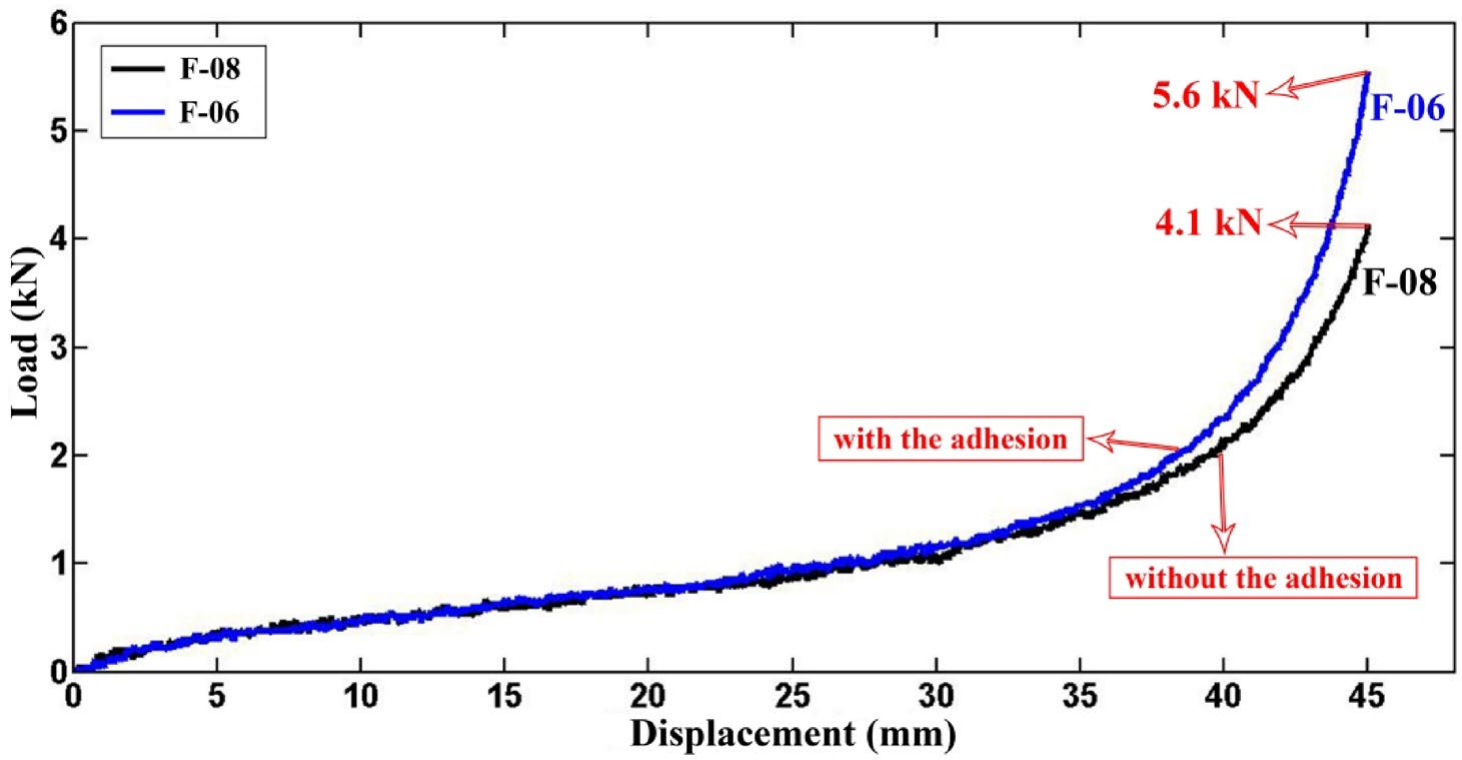
Lateral load–displacement curves of the specimens F-06 and F-08 with and without the adhesion.

Figure 20 compares the lateral load–displacement responses of specimens F-06 and F-08, which were identical in geometry and foam density but differed in the adhesion condition at the foam-tube interface. In F-06, natural adhesion developed between the polyurethane foam and the inner wall of the polyethylene tube, whereas in F-08, the foam was inserted without adhesion. The curves coincide over most of the displacement range, indicating little influence of adhesion on the pre-peak response and deformation path. A divergence emerges only near densification: the peak load increases from 4.1 kN (F-08) to 5.6 kN (F-06), a rise of approximately 36 percent, consistent with enhanced interfacial load transfer and suppressed interfacial slip. Despite this higher peak, the absorbed energy changes only marginally-from 42.0 J for F-08 to 44.40 J for F-06 (about 5.7 percent)-showing that adhesion modifies terminal resistance rather than the integral energy-dissipation capacity during flattening. These trends are in good agreement with prior reports that adhesion can elevate peak load while exerting little influence on total energy absorption^27–29^.

### Synthesis of Parameter Effects

The results of this study highlight that the response of polyethylene tubes under lateral compression is a result of the combined effects of geometry, foam filling, and material interaction. Tube length influenced both the magnitude of the peak load and the amount of energy absorption, with shorter tubes generally resisting higher loads, while longer tubes allowed greater overall energy dissipation. Tube diameter exhibited contrasting trends: in empty tubes, smaller diameters provided superior energy absorption, whereas in foam-filled tubes, larger diameters became more advantageous due to the greater foam volume. Wall thickness increased strength and energy absorption in both empty and filled configurations, though its effect was less dominant when foam was present. Foam filling itself produced the most significant improvement, not only increasing absorbed energy but also stabilizing the load–displacement response, with higher-density foams delivering the most efficient performance. Adhesion between the foam and the tube wall further enhanced peak load capacity, though it had little influence on total energy absorption. In addition, the effect of relaxation time was found to be limited, leading only to a uniform reduction in the final diameter of the tubes after unloading, independent of their initial geometry.

## Conclusions

In this study, the lateral compression response of circular polyethylene tubes was systematically investigated by comparing empty and polyurethane foam-filled specimens, with emphasis on the influence of tube geometry, foam density, and adhesion. A direct relationship between tube length and performance was established: empty specimen A-01 (L = 40 mm) carried 0.64 kN and absorbed 3.315 J, while A-03 (L = 30 mm) reached 0.94 kN and 5.162 J, and A-04 (L = 20 mm) attained 1.12 kN and 7.006 J, confirming that shorter tubes resisted higher loads while longer tubes dissipated more energy overall. Among foam-filled tubes, a similar length dependence was observed, with F-05 (L = 63 mm) absorbing 64.563 J, compared with F-06 (L = 30 mm, 33.084 J) and F-07 (L = 31.5 mm, 36.481 J). Tube diameter showed contrasting effects depending on the presence of foam: in empty tubes, the smaller-diameter A-03 (D = 20 mm) sustained 1.4 kN and absorbed 6.78 J, outperforming the larger-diameter B-03 (D = 25 mm, 1.1 kN, lower energy absorption), whereas in foam-filled tubes the larger-diameter B-02 (D = 25 mm) sustained 48 kN and absorbed 167 J, compared with A-02 (D = 20 mm, 30 kN and 59 J). Increasing wall thickness enhanced energy absorption in empty tubes, with E-03 (t = 3 mm) absorbing 10.5 J compared to F-03 (t = 5 mm, 25.5 J), but the effect was even more pronounced in foam-filled tubes: E-02 (t = 3 mm) absorbed 363 J at a peak load of 38 kN, while F-02 (t = 5 mm) absorbed 473 J at 52.5 kN, highlighting that foam filling combined with thicker walls provides superior resistance. More importantly, filling tubes with foam was far more effective than wall thickening alone: foam-filled F-02 absorbed 488.34 J with a specific absorbed energy (SAE) of 11.44 J/kg, compared to empty F-03, which absorbed only 26.83 J with an SAE of 1.13 J/kg, representing increases by factors of 18 and 10, respectively. Foam density also played a critical role, as E-02 with high-density foam (256 kg/m3) reached 33 kN, outperforming E-05 with medium-density foam (84.8 kg/m3, 6 kN) and E-06 with low-density foam (55.6 kg/m3, 4.5 kN). Adhesion between foam and tube wall increased peak load capacity by 36 percent, with F-06 (with adhesion) reaching 5.6 kN and 44.4 J, compared to F-08 (without adhesion, 4.1 kN and 42.0 J), while the total absorbed energy changed only slightly. Finally, after relaxation, all specimens showed a consistent diameter reduction of 12.7 percent, independent of geometry. Overall, the results demonstrate that polyurethane foam filling, especially with higher-density foams and strong adhesion, markedly enhances the crashworthiness of polyethylene tubes, and that the effects of geometry differ fundamentally between empty and filled tubes, providing quantitative design guidance for optimizing energy absorption and structural efficiency in structural engineering applications.

## Data Availability

The experimental datasets analyzed during the current study are available from the corresponding author upon request.
